# Corrigendum: Direct Observation of Long Electron-Hole Diffusion Distance in CH_3_NH_3_PbI_3_ Perovskite Thin Film

**DOI:** 10.1038/srep20265

**Published:** 2016-02-02

**Authors:** Yu Li, Weibo Yan, Yunlong Li, Shufeng Wang, Wei Wang, Zuqiang Bian, Lixin Xiao, Qihuang Gong

Scientific Reports 5: Article number: 1448510.1038/srep14485; published online: 09292015; updated: 02022016

The original version of this Article contained a typographical error in the Abstract.

“Short diffusion distance of few hundreds of nanosecond was also observed in thin films.”

now reads:

“Short diffusion distance of few hundreds of nanometer was also observed in thin films.”

This has now been corrected in the PDF and HTML versions of the Article.

