# Supplementary material for: Direct Observation of Long Electron-Hole Diffusion Distance in CH3NH3PbI3 Perovskite Thin Film
Source: Sci Rep. 2015 Sep 29;5:14485. doi: 10.1038/srep14485 (PMC4586438; doi:10.1038/srep14485)
Supplement: Supplementary Information [file srep14485-s1.pdf]

SUPPLEMENTARY INFORMATION

Direct Observation of Long Electron-Hole Diffusion Distance in $CH_3NH_3PbI_3$ Perovskite Thin Film

Yu Li[1,§], *Weibo Yan*[2,§], *Yunlong Li*[2], *Shufeng Wang*[1,*], *Wei Wang*[1], *Zuqiang Bian*[2,*], *Lixin Xiao*[1], *and Qihuang Gong*[1]

[1]Institute of Modern Optics & State Key Laboratory for Artificial Microstructure and Mesoscopic Physics, School of Physics, Peking University, Beijing 100871, China.

[2]State Key Laboratory of Rare Earth Materials Chemistry and Applications, College of Chemistry and Molecular Engineering, Peking University, Beijing, 100871, China

[§]These authors contributed equally to this work. *Correspondence and requests for materials should be addressed to S.W. (email: wangsf@pku.edu.cn) or to Z.B. (email: bianzq@pku.edu.cn)

Supplementary Methods

Calculation of absorption coefficient. The absorption (*A*) was calculated using:

$$A = -log(\frac{I_s}{I_0}) \quad \text{(S1)}$$

where I_0 is the reference intensity measured with the blank and I_s is the intensity measured with the sample.

The absorption coefficient α was obtained from:

$$T = \frac{I_s}{I_0} = exp(-\alpha L) \quad \text{(S2)}$$

where *T* represents transmittance and *L* is the sample thickness.

From Equation S1 and S2, we can get the absorption coefficient by the value of *A*:

$$\alpha = A \cdot ln10/L \quad \text{(S3)}$$

The absorption coefficient for $CH_3NH_3PbI_3$ is as a function of wavelength. The penetration depth $\tau(\lambda)$ is then calculated as $\tau(\lambda) = 1/\alpha(\lambda)$.

Calculation of charge diffusion coefficient and diffusion length.

The charge diffusion length was calculated based on one-dimensional diffusion model which was described in Xing's paper (Xing, G. *et al. Science* **342,** 344-347 (2013)) as below:

$$N(t) = \frac{2n_0 L}{\pi} exp(-k(t)t) \sum_{m=0}^{\infty}(exp\left(-\frac{\pi^2 D}{L^2}\left(m+\frac{1}{2}\right)^2 t\right)\frac{exp(-\alpha L)\pi\left(m+\frac{1}{2}\right)+(-1)^m \alpha L}{\left((\alpha L)^2+\pi^2\left(m+\frac{1}{2}\right)^2\right)\left(m+\frac{1}{2}\right)}) \quad \text{(S4)}$$

where $N(t)$ is overall photocarrier density within perovskite film, D is the diffusion coefficient, n_0 is initial photoinduced carrier distribution, $k(t)$ is the observed PL decay rate without any quencher layers, α is the absorption coefficient, and L is the thickness of perovskite layer.

By fitting time-resolved PL results we can obtain the expression of $k(t)$. PL decay curves in our experiments are fitted by stretched exponential model or simple rate equation.

For stretched exponential fitting:

$$I(t) = I_0 e^{-(t/\tau_s)^{\beta}} \quad \text{(S5)}$$

From Equation (S5) we can obtain the expression of $k(t) = \beta \tau_s^{-\beta} t^{\beta-1}$.

For pristine films with mono- and bi-molecular behavior, we use simple rate equation:

$$-\frac{dn}{dt} = An + Bn^2 \quad \text{(S6)}$$

$$I_{PL} \propto Bn^2 + BNn \quad \text{(S7)}$$

where n is the photogenerated carrier density, A is the monomolecular trapping rate, B is the bimolecular radiative recombination coefficient and N is the emissive trap density. The effective PL lifetime then can be written as

$$\tau_{PL} = (A + Bn_0)^{-1} \quad \text{(S8)}$$

From Equation (S8) we obtain $k(t) = (A + Bn_0)$. Sequentially $N(t)$ is used to fit the PL decay of perovskite films coated with quenchers to derive the value of D. It is notable that the observed

PL intensity $I(t)$ is the convolution of the intrinsic PL intensity $f(t)$ and the instrument response function (IRF) $g(t)$:

$$I(t) = \int g(t) f(t - t') dt' \tag{S9}$$

So by fitting the observed PL decay with the convolution of IRF gives the estimated D value. Finally the charge carrier diffusion length L_D is calculated by $L_D = \sqrt{D\tau_{PL}}$, where τ_{PL} is the fitted PL lifetime in the case of no quenching layers.

Supplementary Figure 1.

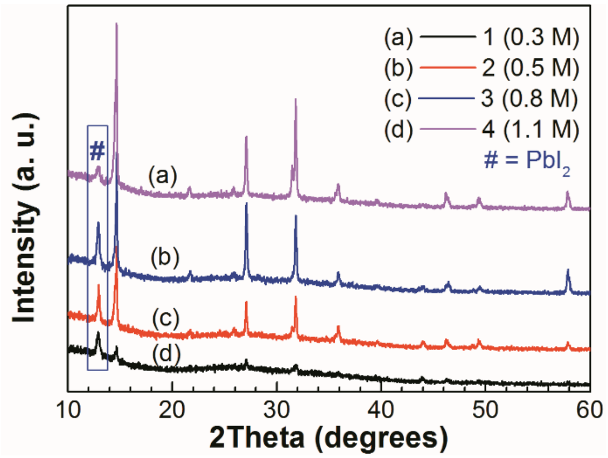

Supplementary Figure 1. XRD patterns of $CH_3NH_3PbI_3$ films fabricated with varied PbI2 concentration: (**a**) 0.3 M, (**b**) 0.5 M, (**c**) 0.8 M, and (**d**) 1.1 M, with corresponding thickness of 63, 156, 254, and 310 nm.

Supplementary Figure 2.

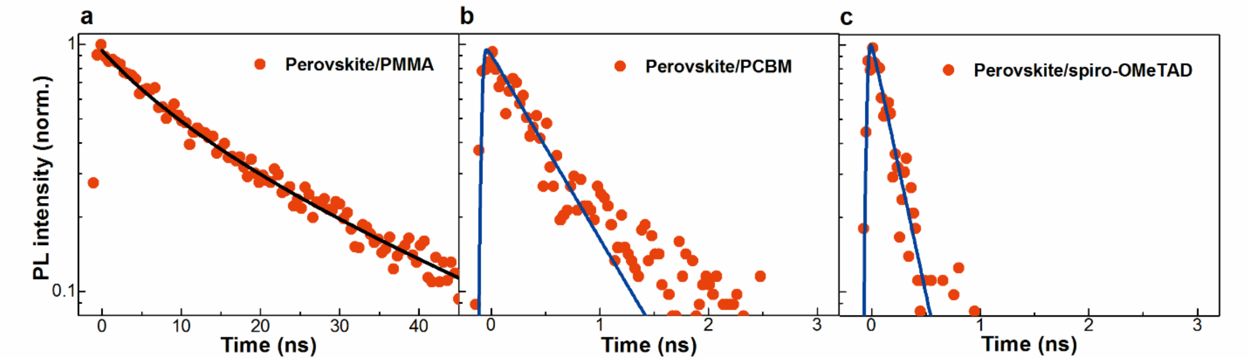

Supplementary Figure 2. PL decays (red circles) of $CH_3NH_3PbI_3$ perovskite (ca. 95 nm) coated with (**a**) PMMA, (**b**) PCBM and (**c**) Spiro-OMeTAD layer, taken at the peak emission wavelength, excited at 517 nm, 90 nJ/cm^2. The black (a) and blue solid lines (b and c) are the fits to the PL results by using rate equation and one-dimensional diffusion model, respectively.

Supplementary Figure 3.

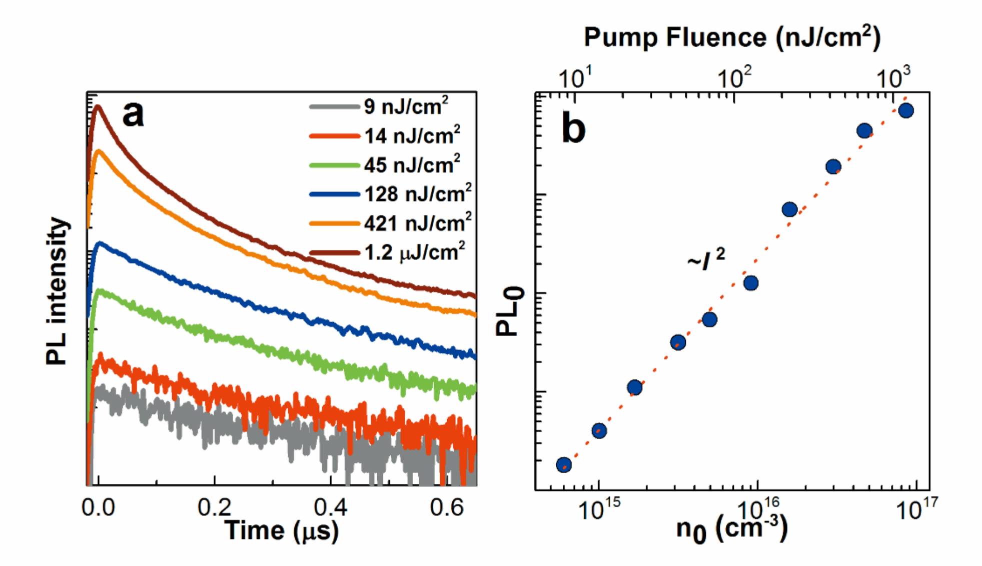

Supplementary Figure 3. Time-resolved photoluminescence spectroscopy and their pump energy dependency. (a) Pump fluence dependent PL decays of a thick $CH_3NH_3PbI_3$ perovskite (ca. 370 nm) thin film, excited at 517 nm. (b) Photoluminescence emission intensity at t=0 (PL_0) as a function of photocarrier density (lower axis) and laser pump fluence (upper axis).

Supplementary Figure 4.

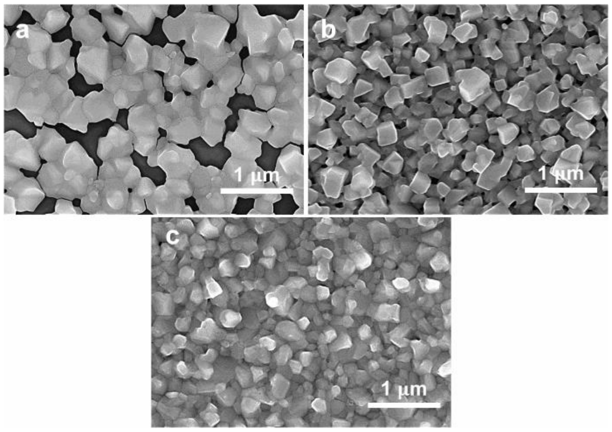

Supplementary Figure 4. SEM images of a series of thick perovskite film containing crystal grains of different sizes. Top-view SEM images of $CH_3NH_3PbI_3$ deposited via changing CH_3NH_3I concentration: (**a**) 10 mg/mL; (**b**) 15 mg/mL; (**c**) 20 mg/mL.

Supplementary Figure 5.

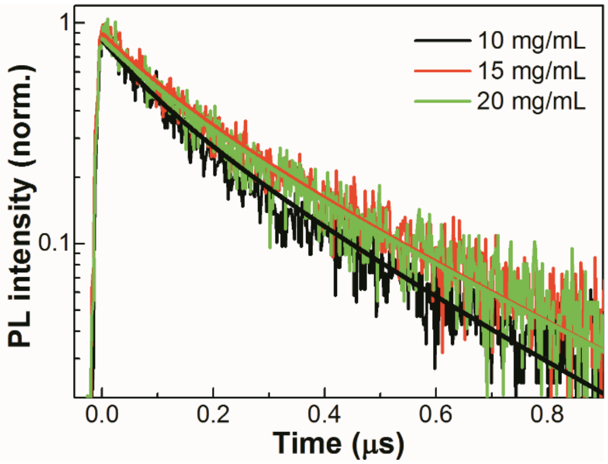

Supplementary Figure 5. PL decay curves of $CH_3NH_3PbI_3$ thin films made by various CH_3NH_3I concentration upon excitation at 517 nm, 24 nJ/cm^2. The solid lines are the rate equation fits to the corresponding results.

Supplementary Figure 6.

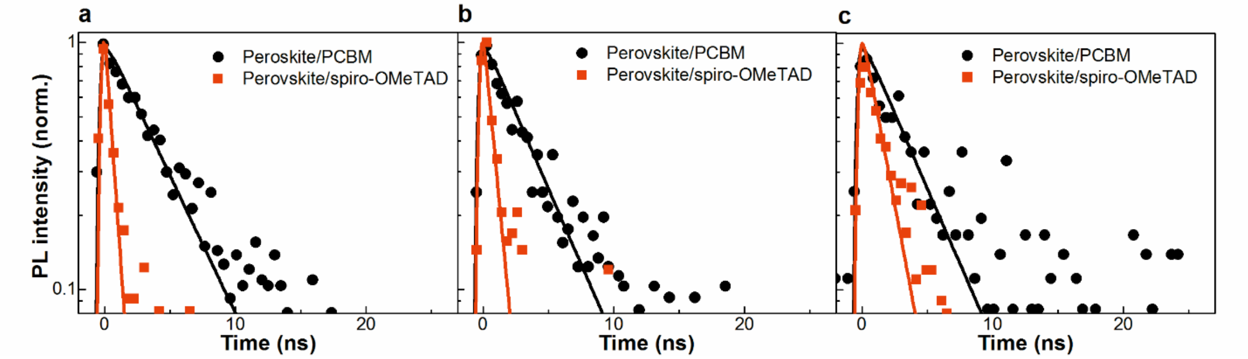

Supplementary Figure 6. PL decay curves of $CH_3NH_3PbI_3$ thin films coated with an electron (PCBM; black circles) or hole (Spiro-OMeTAD; red squares) quenching layer upon excitation at 517 nm, 24 nJ/cm^2. $CH_3NH_3PbI_3$ thin films are formed using varied CH_3NH_3I concentrations shown in SI Figure 4 &5: (**a**) 10 mg/mL; (**b**) 15 mg/mL; (**c**) 20 mg/mL. The solid lines are the one-dimensional model fits to the corresponding results.

Supplementary Table 1. Summary of the thickness (L), the fitted monomolecular trapping rate (A), bimolecular radiative recombination coefficient (B), the trap density (N) and the effective PL lifetime (τ_{PL}) in Supplementary Figure 5. The lifetime of perovskite coated with electron transport layer (ETL) or hole transport layer (HTL), the calculated diffusion coefficients (D), and diffusion lengths (LD) in Supplementary Figure 6 are also presented.

CH_3NH_3I (mg/mL)	L (nm)	A (s^{-1})	B (cm^3/s)	N (cm^{-3})	τ_{PL} (ns)	$\tau_{w/ETL}$ (ns)	$\tau_{w/HTL}$ (ns)	Species	D (cm^2/s)	L_D (μm)
10	~345	3.2×10^{6}	1.2×10^{-9}	4.0×10^{15}	189.0	3.1	0.57	Electrons	0.12	1.5
								Holes	0.85	4.0
15	~370	2.9×10^{6}	0.9×10^{-9}	3.7×10^{15}	230.0	2.8	0.63	Electrons	0.15	1.7
								Holes	0.70	4.0
20	~390	2.8×10^{6}	1.0×10^{-9}	4.4×10^{15}	222.8	2.5	1.2	Electrons	0.17	1.9
								Holes	0.40	3.0

Supplementary Table 2. Pump fluence dependent PL lifetime. The effective PL lifetime for $CH_3NH_3PbI_3$ thin films fabricated by using 1.0 M PbI_2 and various concentration of CH_3NH_3I solution (10 mg/mL, 15 mg/mL, and 20 mg/mL).

**Pump Fluence (nJ/cm^2)**	**τ_{PL} (ns)**		
	**10 mg/mL**	**15 mg/mL**	**20 mg/mL**
9	254.8	295.5	293.4
14	225.4	266.9	262.3
24	189.0	230.0	222.8
45	139.7	176.4	167.6
70	105.7	137.1	128.4
